# Assessing Prokaryotic Benthic Communities in the Red Sea

**DOI:** 10.1111/1462-2920.70216

**Published:** 2026-02-21

**Authors:** Christopher A. Hempel, Larissa Frühe, Sofia Frappi, Elisa Laiolo, Kah Kheng Lim, Diego E. Rivera Rosas, Amal A. Bajaffer, Wajitha J. R. M. Sait, Alexandra Steckbauer, Taiba Alamoudi, Jacqueline V. Alva García, Shannon G. Klein, Anieka J. Parry, Mohammad A. Qurban, Vincent A. Pieribone, Carlos Angulo‐Preckler, Carlos M. Duarte

**Affiliations:** ^1^ Marine Science Program, Biological and Environmental Science and Engineering Division (BESE) King Abdullah University of Science and Technology (KAUST) Thuwal Kingdom of Saudi Arabia; ^2^ OceanX New York New York USA; ^3^ National Center for Wildlife (NCW) Riyadh Kingdom of Saudi Arabia

## Abstract

Marine sediments host diverse benthic prokaryotic communities that are integral to global biogeochemical cycles. However, the spatial distribution and environmental drivers of these communities, particularly in unique environments like the Red Sea, remain largely underexplored. In this study, we examine benthic prokaryotic communities sampled during the Red Sea Decade Expedition (RSDE) using 16S rRNA gene sequencing across five major regions along the Red Sea's latitudinal gradient and three depth strata. Our findings reveal distinct biogeographical patterns shaped by depth, latitude, and oxygen availability, with clear shifts in microbial community composition across the epibenthic, mesobenthic and bathybenthic zones. Bathybenthic communities exhibited consistently low levels of OTU richness throughout the Red Sea, likely due to uniform niche environmental conditions at depth, while shallower communities showed higher OTU richness towards the Southern Red Sea. The southern region harboured higher relative abundances of Chloroflexi and reduced relative abundances of Proteobacteria and Acidobacteriota relative to the northern regions. Extreme environments, such as the Atlantis II brine pool, supported specialised microbial communities likely adapted to extreme conditions like hypersalinity. This study established a critical baseline for understanding the responses of marine microbial communities to climate change and their roles in biogeochemical processes.

## Graphical abstract

Prokaryotic benthic communities in the Red Sea are shaped by depth, latitude, and oxygen availability. Deep‐sea communities exhibited low variance in OTU richness, while shallower communities showed higher OTU richness towards the Southern Red Sea. Extreme environments supported specialised microbial communities likely adapted to extreme conditions like hypersalinity.

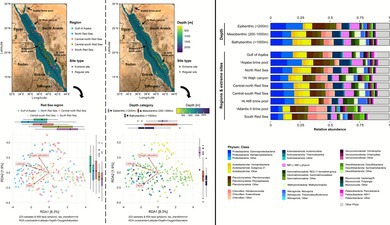

## 1 Introduction

Marine sediments cover approximately 70% of the Earth's surface, yet the taxonomic diversity and spatial distribution of benthic microbial communities remain far less understood than those in pelagic waters (Laiolo et al. 2024). As the largest reservoir of organic carbon on the planet (Wellsbury and Parkes 2000), marine sediments play a crucial role in the marine ecosystem, supporting a vast diversity of benthic microorganisms (Kallmeyer et al. 2012; Parkes et al. 2014). These microorganisms drive essential global biogeochemical processes, including nutrient cycling, carbon sequestration, and biogeochemical transformations crucial for maintaining short‐ and long‐term ecosystem balance (Fuhrman 2009; Nogales et al. 2011).

While the ecological significance of benthic microorganisms is well recognised (Ruff et al. 2024; Teske 2013), many aspects of their spatial distribution and environmental constraints remain insufficiently characterised, especially for the deep sea. This is partly due to the challenges of obtaining microbiological samples from seafloor sediments at depth. The collection of these samples is often limited by difficult access and high costs, making it far more challenging in contrast to pelagic sampling. Additionally, variations in quality control measures and analytical protocols across different studies further hinder a comprehensive understanding of these microorganisms (Liu et al. 2023; Morono and Inagaki 2016). Despite these limitations, studies employing 16S rRNA gene sequencing have shown that diverse bacterial taxa are ubiquitous in organic‐rich anoxic sediments and that microbial community composition is structured by depth as well as geochemical and sedimentological properties (Inagaki et al. 2015; Parkes et al. 2005; Teske 2013). Different marine geographic regions have also been shown to host distinct benthic microbial communities (Inagaki et al. 2003; Newberry et al. 2004; Rochelle et al. 1994; Webster and Negri 2006). These differences have been attributed to environmental selection via changes in pH, temperature, oxygen and silicate concentration (Gilbert et al. 2009; Hollister et al. 2010; Kirchman et al. 2010) or hydrographic factors like ocean currents and tides, which affect the dispersion of microorganisms (Hamdan et al. 2013). Advances in single‐cell genomics, metagenomics, and functional gene analysis have further illuminated some of the metabolic capabilities of predominant bacterial and archaeal taxa (Lloyd et al. 2013), highlighting the need for a more comprehensive understanding of the spatial distribution and functional dynamics of marine benthic microbial communities.

The Red Sea represents a globally unique ocean basin, characterised by exceptionally high temperatures even in its deepest areas (minimum temperature: 21°C), as well as low oxygen levels (below 1 mg O_2_ L^−1^), low nutrient concentrations, and a high degree of geographical isolation from neighbouring marine regions, including the Mediterranean and Indian Ocean. Consequently, the Red Sea exhibits one of the highest levels of endemism for marine life (DiBattista et al. 2016). While previous studies have explored pelagic microbial diversity in the Red Sea (Calleja et al. 2018; Frühe et al. 2025; García et al. 2018; Ngugi et al. 2012; Pearman et al. 2017), research on benthic microbial communities beyond shallow coral reefs remains limited, despite their ecological significance. This situation changed in 2022 with the launch of the Red Sea Decade Expedition (RSDE), a marine expedition surveying the Exclusive Economic Zone of the Kingdom of Saudi Arabia in the eastern Red Sea, aimed at expanding our knowledge of the biodiversity of this unique ecosystem.

Here, we report on the benthic prokaryotic communities sampled during the RSDE, analysed through 16S rRNA gene sequencing. We examined community dynamics across five major geographical regions and three depth categories, resulting in a comprehensive inventory of benthic prokaryotic communities in the Red Sea. Our analyses revealed distinct biogeographical patterns and relationships between the unique characteristics of the Red Sea and its benthic microbial communities. These results are crucial for understanding the ecological dynamics of marine sediments in this distinctive region.

## 2 Material and Methods

### 2.1 Sample Collection

**FIGURE 1:**
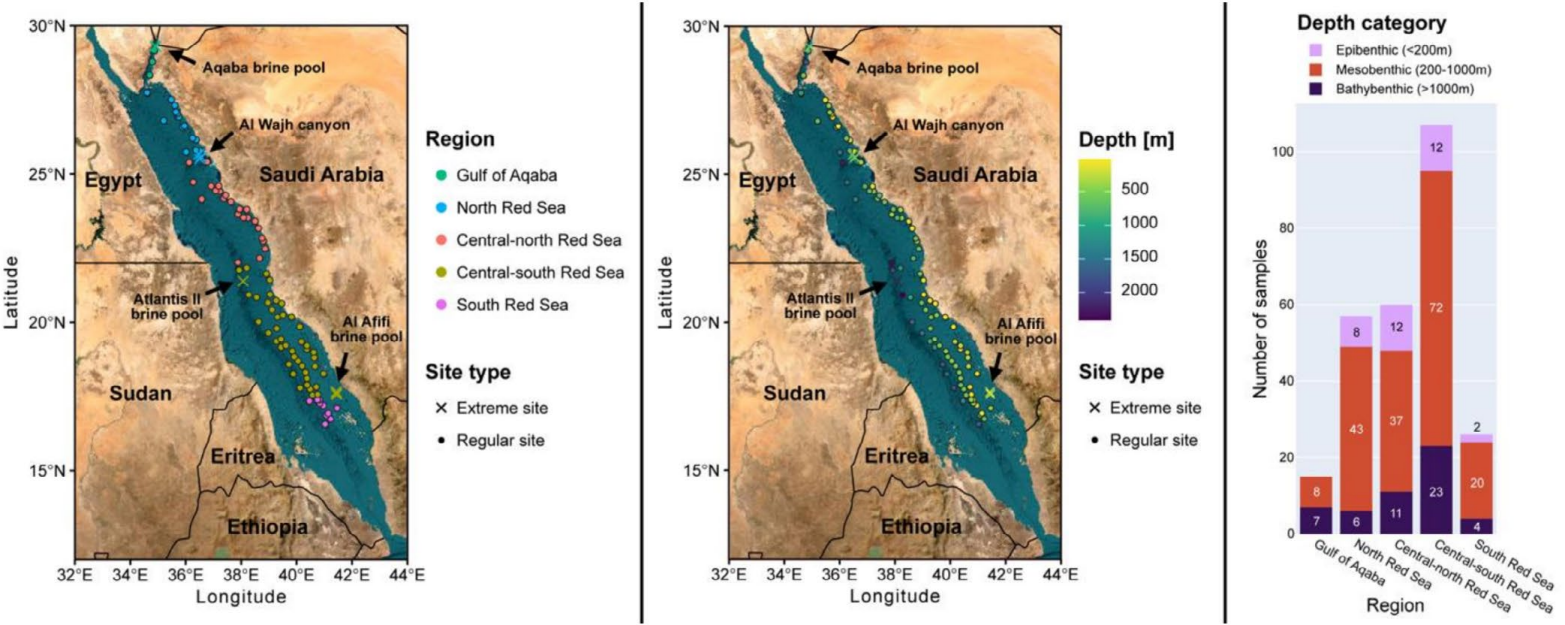
Overview of sampling sites, highlighted by geographical region (left) and depth (middle), as well as the distribution of samples across depth categories (right). Extreme deep‐sea environments are marked with arrows.

Sampling was conducted as part of the Red Sea Decade Expedition (RSDE) from February to June 2022 in the eastern Red Sea, onboard the research vessel OceanXplorer. The detailed expedition setup is explained in Frühe et al. (2025). In summary, 265 sediment samples were collected within the Exclusive Economic Zone of the Kingdom of Saudi Arabia, located in the Eastern Red Sea, following a latitudinal and bathymetric gradient (Figure 1). The bathymetric gradient ranged from shallow coastal samples to deep‐sea samples taken along the axial rift valley of the Red Sea, with sediment samples taken at depths ranging from 20–2415 m below sea level. The expedition also included sampling of four unique deep‐sea environments of interest, namely the Atlantis II brine pool, the Al Afifi brine pool, the Aqaba brine pool, and the Al Wajh Canyon (Figure 1). All metadata for all samples can be found in Table S1.

Sediment samples were primarily collected using a custom‐built Argus Mariner XL ROV or manned Triton 3300/3 submersibles, both equipped with robotic arms to operate acrylic push cores (average length: 50 cm) to sample the seabed along a transect at each site. After returning to the surface, supernatant water was carefully drained from the push cores with a sterile syringe and tubing while leaving the sediment undisturbed. Surface sediments (maximum depth: 1 cm) were sampled using sterile spatulas, and three biological replicates (approximately 5 g/replicate) were transferred into sterile 15 mL Falcon tubes. RNAlater Stabilisation Solution (Thermo Fisher Scientific) was added to maintain nucleic acid integrity, followed by storage at +4°C until further processing. Additionally, three shallow samples (< 40 m depth) were collected by scuba divers using Falcon tubes, following the same processing and storage protocols as those used for ROV and submersible‐collected samples. We also added six negative field controls by transferring only RNAlater Stabilisation Solution to Falcon tubes at regular intervals. The tubes were kept open on the deck during core sampling operations.

### 2.2 Environmental Data Collection

Environmental data was collected using an RBR Maestro CTD attached to the Argus Mariner XL ROV to measure temperature, salinity, oxygen availability (percent air saturation), conductivity, pressure, chlorophyll, backscatter, transmittance, speed of sound, and density anomalies. The CTD was attached to the ROV at a height of 1.5 m, which corresponds to the height above seafloor level at which the measurements were taken. Sensor data were processed in 10‐min intervals, with each interval corresponding to the sampling time of the sediment core (±5 min). The mean average, standard deviation (SD), maximum (max), minimum (min) and sample size (*n*) were then calculated for each interval.

Throughout the RSDE, water samples were collected using Niskin bottles mounted to the ROV (*N* = 119, from 42.8–2335.9 m depth). Water samples were analysed via the automated Winkler method (Carpenter 1965; Dickson 1994; Winkler 1888). Briefly, triplicate water samples were carefully syphoned into 120 mL volume‐calibrated borosilicate bottles with stoppers. Bottles were allowed to overflow for at least two volumes (> 240 mL), sealed, and fixed with 1 mL Manganese sulfate monohydrate and 1 mL NaOH/NaI solution before being stored in total darkness for 2 h. After 2 h, 1 mL sulfuric acid (10 N) was added, and the measurements were performed using sodium thiosulfate. Titrations were done using a T50 automatic titrator (Mettler Toledo) configured with an InMotion Pro autosampler (Mettler Toledo; Carpenter 1965; Dickson 1994; Winkler 1888).

The data obtained from the RBR Oxyguard oxygen sensor and Winkler titrations showed a high agreement (*R* ^2^ = 0.95), but we detected negative offsets in the RBR Oxyguard measurements. Given this apparent sensor drift, we aligned the RBR Oxyguard with the calculated average offset for each leg of the campaign. Following the corrections, the RBR Oxyguard and Winkler titrations showed a higher agreement with an *R* ^2^ > 0.96.

### 2.3 Laboratory Processing

DNA was extracted using the PowerSoil Pro Kit (Qiagen) with an optimised version of the manufacturer's protocol. Briefly, about 300 mg of sediment and 800 μL of CD1 buffer were added to the PowerBead Pro tubes and incubated at 4°C overnight. Prior to the bead‐beating step, samples were incubated at 65°C for 10 min. At step 7, 600 μL of CD3 buffer was added, and samples were incubated on ice for 10 min, followed by vortexing for 5 s. At step 15, samples were centrifuged at 17,000 × g for 5 min. These modifications increased DNA yield and amplification rates. Six negative extraction controls were processed alongside true samples using only the extraction kit reagents, omitting any sediment input.

The concentration of extracted DNA was determined using the dsDNA HS (High Sensitivity) Assay Kit and a Qubit Fluorometer (ThermoFisher Scientific). DNA was amplified targeting the V3–V4 region of the 16S SSU rRNA gene using the 341F/805R primer set (Herlemann et al. 2011) with the following PCR thermal protocol: (1) an initial denaturation step at 98°C for 2 min, followed by 28 cycles of 98°C for 20 s, 54°C for 20 s, and 72°C for 15 s, with a final elongation step at 72°C for 2 min. The PCR reaction mix comprised 5 μL KAPA 2× Master Mix (Roche), 3.25 μL of PCR‐grade water, 1 μL of 5 mM forward primer, 1 μL of 5 mM reverse primer and 0.25 μL of 20 mg/mL BSA (ThermoFisher Scientific). For each reaction, 1 μL of DNA template was used. Six negative PCR controls were also processed by adding 1 μL of Milli‐Q water (MilliporeSigma) to the PCR reaction mix instead of the DNA template. PCRs were run in triplicates to reduce PCR‐related bias, and successful amplification was verified via gel electrophoresis.

The three technical replicates were then pooled and cleaned using AMPure XP Beads (Beckman Coulter). Sequencing libraries were prepared using the Illumina Nextera XT kit, cleaned using AMPure XP Beads, and then sequenced on an Illumina NovaSeq6000 (2 × 250 bp) at King Abdullah University of Science and Technology (KAUST) Sequencing Core Labs.

### 2.4 Bioinformatics Processing

Demultiplexing was carried out by the sequencing facility. Paired‐end reads were processed with a modified version of Apscale v1.6.3 (Buchner et al. 2022) in which we implemented Swarm (Mahé et al. 2022) as an alternative to vsearch (Rognes et al. 2016) for Operational Taxonomic Unit (OTU) clustering, the option to first denoise reads and then cluster the denoised reads into OTUs and microDecon (McKnight et al. 2019) to remove reads found in negative field, extraction, and PCR controls from true samples.

We ran the modified Apscale pipeline with the following parameters: maxEE was set to 2, which discarded reads with more than 2 expected errors, the minimum and maximum read lengths were set to 350 and 550 bp, which discarded merged reads that were not within the target amplicon length, reads were first denoised with unoise using default parameters and then clustered into OTUs using Swarm with default parameters (including local clustering threshold *d* = 1), and default parameters were used for paired‐end merging, primer trimming, dereplication, pooling, and LULU (Frøslev et al. 2017) filtering. We processed the sequences into OTUs rather than Amplicon Sequence Variants (ASVs) since ASVs may not accurately reflect species composition (Hakimzadeh et al. 2024). The modified version of Apscale can be found on GitHub (https://github.com/hempelc/apscale), as well as a wrapper to run Apscale in the command line (https://github.com/hempelc/apscale_wrapper). The denoised reads and processed OTUs are available as ASV and OTU tables, both before/after LULU‐based post‐curation and before/after microDecon‐based negative control removal, in Supporting Information S1.

We downloaded the DADA2‐formatted SILVA NR99 database version 138.1 (10.5281/zenodo.4587954; Callahan et al. 2016; McLaren and Callahan 2021; Quast et al. 2013) and assigned taxonomy to the generated OTUs using BLAST (Altschul et al. 1990) with an E‐value threshold of 1e−05. We filtered BLAST hits as follows: (1) all hits with a bitscore < 150 and an alignment length of < 100 were excluded, (2) for every OTU, all hits whose bitscore did not fall within a 2% margin of the highest bitscore were excluded, (3) if multiple taxa occurred among the remaining hits, only their Lowest Common Ancestor was retained and (4) taxonomic lineages were trimmed based on percentage identity to the query; specifically, we trimmed at 98%, 95%, 90%, 85%, 80% and 75% for the species, genus, family, order, class, and phylum ranks, respectively, meaning that only hits with a percentage identity score of > 98% were assigned to the species level and so forth. This stringent filtering approach ensured the minimization of false‐positive detections.

### 2.5 Further Data Processing and Analysis

OTU tables and environmental variables were further processed in R v4.3.2 (R Core Team 2016) and involved the R packages vegan v2.6.4 (Oksanen et al. 2016), phyloseq v1.46.0 (McMurdie and Holmes 2013), metagMisc v0.5.0 (https://github.com/vmikk/metagMisc), and microViz v0.12.1 (Barnett et al. 2021). Low‐prevalent (prevalence < 3) OTUs were discarded. All analyses were performed using clr‐transformed abundance data following recommendations by Gloor et al. (2017). Samples were categorised into five distinct geographical regions within the Red Sea (Gulf of Aqaba, Northern Red Sea, North‐Central Red Sea, South‐Central Red Sea, and Southern Red Sea) based on latitudinal coordinates following the differentiation proposed by Raitsos et al. (2013) and into three depth categories (epibenthic: < 200 m, mesobenthic: 200–1000 m and bathybenthic: > 1000 m) according to the seafloor depth at which the sediment samples were collected. We performed Redundancy Analysis (RDA) on clr‐transformed, aggregated phyla abundances to examine the relationship between microbial communities and environmental variables, employing forward selection with the ‘ordiR2step’ function of the vegan R package to iteratively add significant environmental variables and maximise explained variance. Furthermore, we investigated correlations between clr‐transformed, aggregated phyla abundances and environmental variables by using the ‘cor_heatmap’ function of the microViz R package, which returns a heatmap of Pearson correlation coefficients. Notably, since oxygen saturation was only available for samples collected with the Argus Mariner XL ROV, the latter two analyses were based on a subset of the samples (*N* = 223), excluding the fourth sampled extreme‐environment ecosystem Atlantis II brine pool. Lastly, to assess the relationship between latitude and OTU richness across different depth zones, we fitted separate linear regression models for each depth category with OTU richness as the response variable and latitude as the predictor variable. For each depth category, we calculated the *p*‐value for the latitude coefficient and visualised regression lines with 95% confidence intervals. All utilised code is available on GitHub (https://github.com/hempelc/rsde_benthic_prokaryotes).

## 3 Results

### 3.1 Benthic Microbial Community Composition

16S rRNA gene sequencing yielded a total of 413,341,655 raw reads (*N* = 265 samples + 18 negative controls; min: 16,365; max: 23,652,002; median average: 1,003,870 reads, SD: 2,299,413.67). Following bioinformatics processing, we retained 318,974,884 high‐quality reads across 342,526 OTUs. Plotting the number of OTUs against the number of reads per sample showed that the number of OTUs did not increase notably past the sequencing depth median of around 1 M reads (Figure S1), indicating that samples were sufficiently sequenced on average. Despite considerable variation in per‐sample sequencing depth (Figure S1), Red Sea regions were sequenced at a relatively even depth, with the exception of the Central‐south Red Sea (Figure S2). Furthermore, sequencing depth was skewed towards deeper samples in regard to depth categories (Figure S3). These variations in sequencing depth can influence analytical results, which is addressed in the discussion.

**FIGURE 2:**
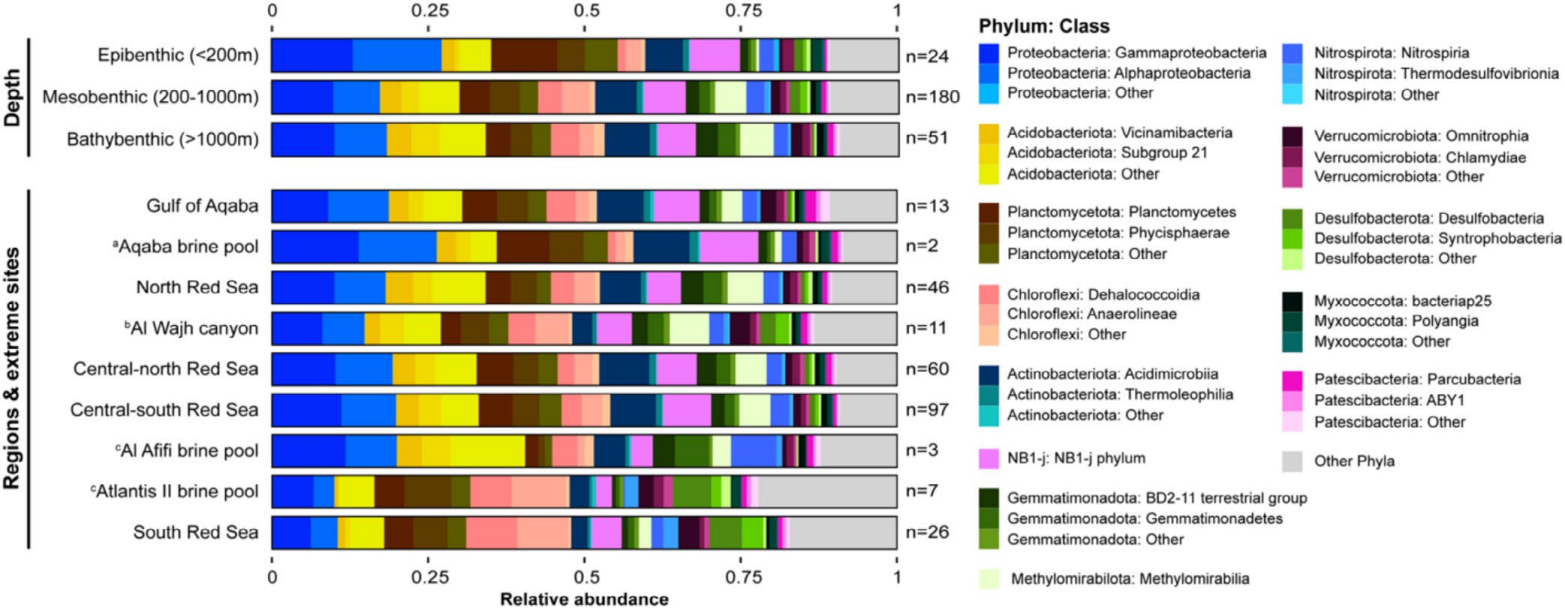
Relative abundances of the 12 most abundant phyla and two most abundant classes within each phylum, aggregated by depth categories (top) and regions & extreme‐environment sites (bottom). The remaining phyla and classes per phylum are grouped under ‘other’. For each group, we calculated the average relative abundances across all samples, and the number of samples per group is denoted on the right side of the bars. Extreme‐environment sites are annotated with superscripted letters. (a) Located within the Gulf of Aqaba, (b) Located within the Northern Red Sea, (c) Located within the South‐Central Red Sea.

54,913 OTUs (16%) were removed via microDecon, and of the remaining 287,613 OTUs, 32,530 (11.3%) were assigned to the genus level. The maximum number of OTUs detected at a single site after microDecon treatment was 98,600, with a median average of 26,228 OTUs per site. The most abundant phyla were Proteobacteria, Acidobacteriota, Planctomycetota and Chloroflexi (Figure 2), comprising 18.2%, 13.5%, 12.4% and 8.4% of total read abundance, respectively. The three most abundant OTUs were assigned to the family Methylomirabilaceae (phylum Methylomirabilota; 1.28% of all reads), the order Actinomarinales (phylum Actinobacteriota; 0.84% of all reads), and the genus *Nitrospira* (phylum Nitrospirota; 1.77% of all reads).

Communities from sunlit seafloors in the epibenthic zone exhibited distinct differences from those at deeper sites, with greater abundances of Proteobacteria in the epibenthic layer (Figure 2, top). Acidobacteriota and Chloroflexi were less abundant in epibenthic samples, while Planctomycetota were more prevalent. Generally, mesobenthic and bathybenthic samples displayed similar community patterns and Methylomirabiliota were nearly absent in epibenthic samples.

The Southern Red Sea was distinctly different from the other geographical regions, characterised by a higher abundance of Chloroflexi and lower abundances of Proteobacteria and Acidobacteriota (Figure 2, bottom). Both the Southern Red Sea and the Atlantis II brine pool showed the highest abundances of Desulfobacterota and were very similar in their community compositions. Other extreme environments showed slightly differing community compositions from their surrounding geographical regions.

## 4 Drivers of Benthic Microbial Community Composition

### 4.1 Redundancy Analysis

**FIGURE 3:**
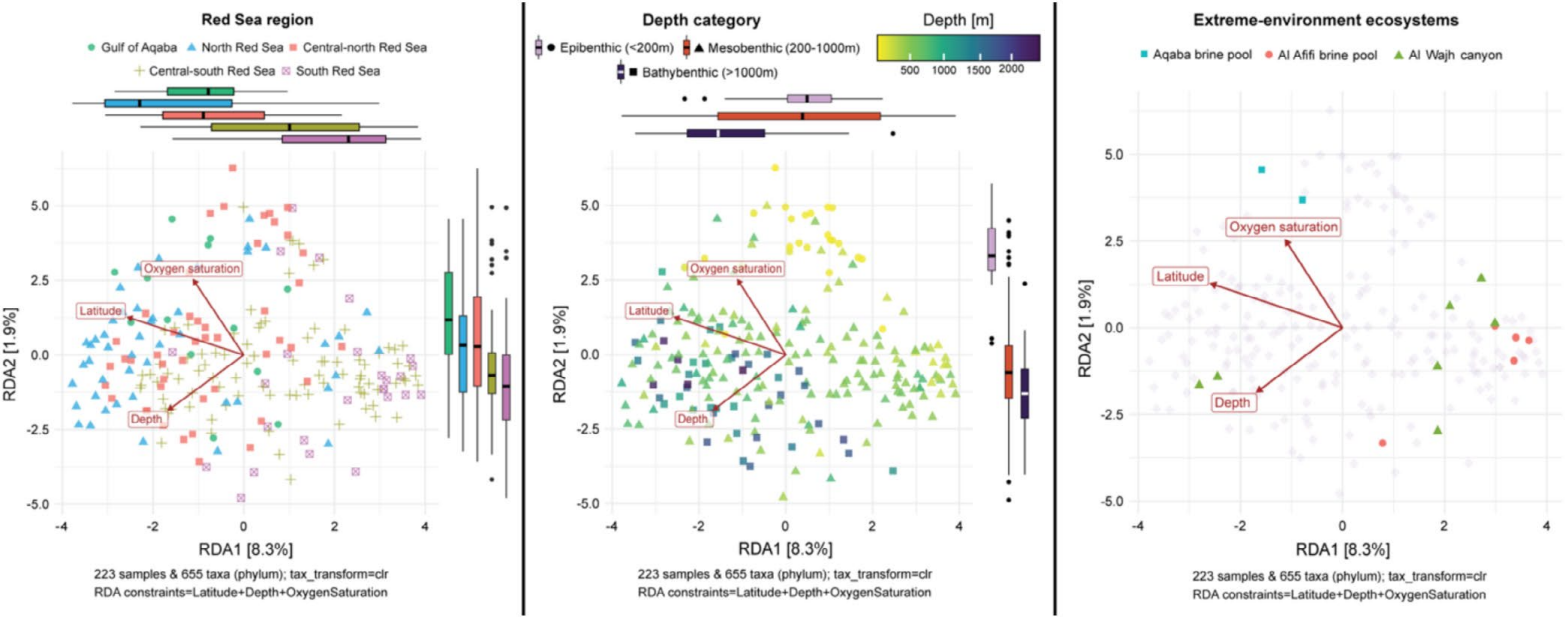
Redundancy analysis (RDA) of clr‐transformed, aggregated phyla abundances of microbial communities, constrained by latitude, depth and oxygen saturation. Left: Samples are coloured according to Red Sea regions. Boxplots represent the distribution of regions on the *x*‐ and *y*‐axes. Middle: Samples are coloured according to depth. Boxplots represent the distribution of depth categories on the *x*‐ and *y*‐axes. Right: Extreme‐environment sites are highlighted, while other sites are shown faintly in the background. Since oxygen saturation was only available for samples collected with the Argus Mariner XL ROV, the RDA is based on a subset of the samples (*n* = 223), excluding the fourth sampled extreme‐environment ecosystem Atlantis II brine pool.

Based on redundancy analysis (RDA) combined with forward selection of environmental variables, the composition of benthic microbial communities was significantly associated with latitude, depth, and oxygen availability. Community compositions were clearly stratified from north to south (Figure 3, left) and along sampling depth (Figure 3, middle), with communities showing greater similarity within each region and depth zone than between different regions.

Among the extreme‐environment ecosystems with available data for oxygen availability, the Al Wajh canyon, located in the Northern Red Sea, and the Al Afifi brine pool, located in the South‐Central Red Sea, showed similarities in community composition (Figure 3, right). Despite their distinct environmental conditions, extreme‐environment ecosystems clustered closely with other benthic communities outside these sites.

### 4.2 Correlation Between Bacterial Phyla Abundance and Environmental Variables

**FIGURE 4:**
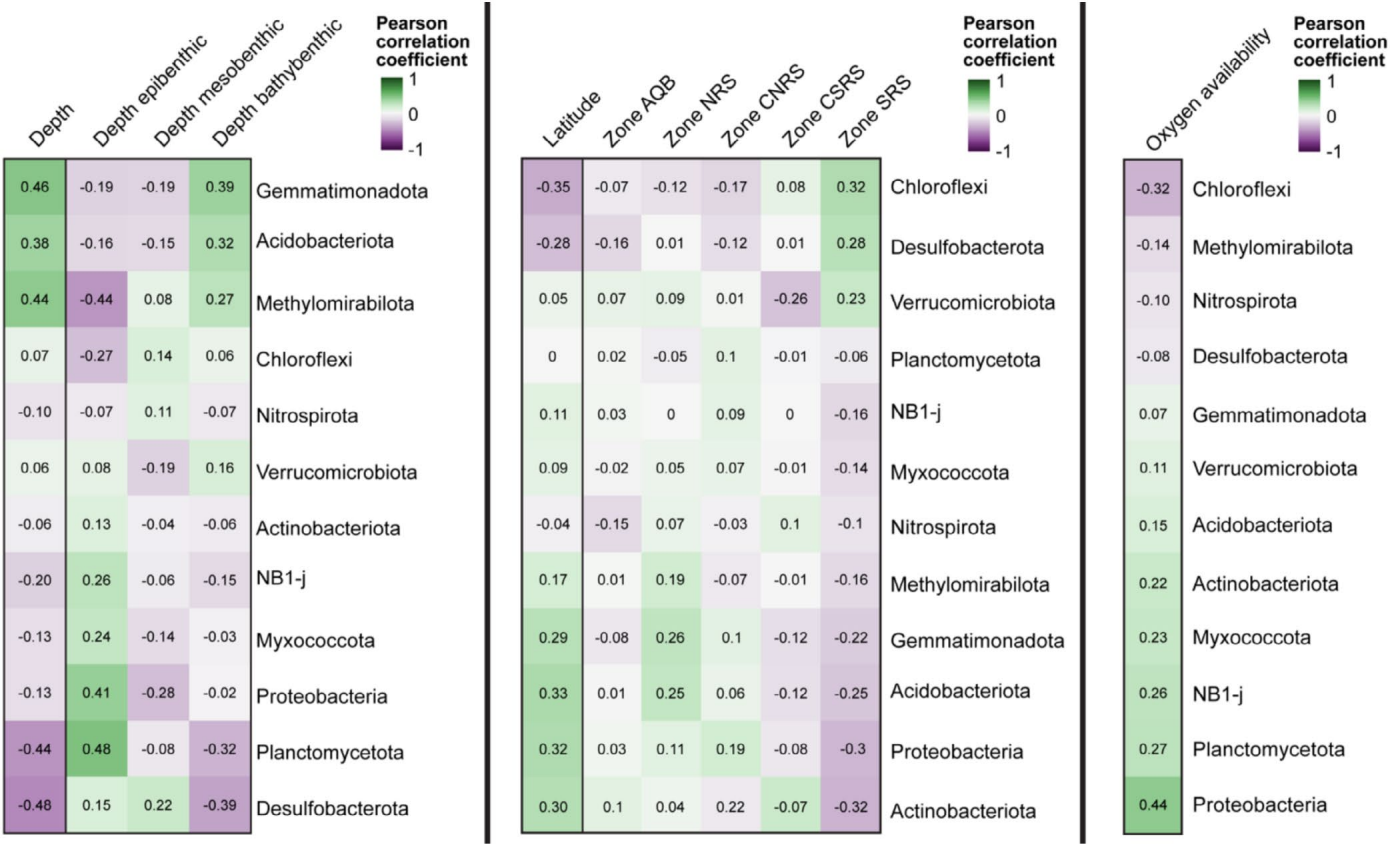
Pearson correlation coefficient heatmaps between clr‐transformed, aggregated phyla abundances of the 12 most abundant phyla and depth categories (left), geographical location (middle), and oxygen availability (right). For depth categories and geographical location, the leftmost columns represent continuous variables, while the remaining columns represent categorical variables derived from the continuous variables. Since oxygen saturation was only available for samples collected with the Argus Mariner XL ROV, the respective correlation coefficients are based on a subset of the samples (*n* = 223) while the remaining coefficients are based on all samples (*n* = 265). AQB, Gulf of Aqaba; NCRS, North‐Central Red Sea; NRS, Northern Red Sea; SCRS, South‐Central Red Sea; SRS, Southern Red Sea.

Among the 12 most abundant phyla, the relative abundance of Planctomycetota and Desulfobacteriota decreased with depth (*r* = −0.44 and −048, respectively), while Gemmatimonadota, Acidobacteriota and Methylomirabilota increased in relative abundance with depth (*r* = 0.46, 0.38, and 0.44, respectively) (Figure 4, left). Overall, we could not detect significant correlations of other phyla with depth. However, within individual depth zones, Proteobacteria were more associated with the epibenthic zone (*r* = 0.41) and less associated with the mesobenthic zone (*r* = −0.28), Chloroflexi were less relatively abundant in the epibenthic zone (*r* = −0.27) and NB1‐j and Myxococcota were more relatively abundant in the epibenthic zone (*r* = 0.26 and 0.24, respectively). Correlations of other phyla within individual depth zones followed the trend of depth overall.

Chloroflexi and Desulfobacterota increased in relative abundance southwards (*r* = −0.35 and −0.28, respectively), while Gemmatimonadota, Acidobacteriota, Proteobacteria and Actinobacteriota increased in relative abundance northwards (*r* = 0.29, 0.33, 0.32 and 0.30, respectively) (Figure 4, middle). Notably, the Southern Red Sea strongly impacted the correlation between phyla and latitude.

Chloroflexi were also correlated to lower oxygen availability (*r* = −0.32), and Proteobacteria correlated with higher oxygen availability (*r* = 0.44) (Figure 4, right).

Overall, Gemmatimonadota and Acidobacteriota showed higher relative abundances at deeper sites and higher latitudes, whereas Desulfobacterota were more prevalent in shallower sites and at lower latitudes. This distinction makes them key phyla for differentiating benthic spatial regions within the Red Sea. Chloroflexi and Proteobacteria also represented key phyla for distinguishing benthic regions according to oxygen availability, while also being strongly driven by latitude.

### 4.3 Latitudinal and Bathymetric Trends in OTU Richness

**FIGURE 5:**
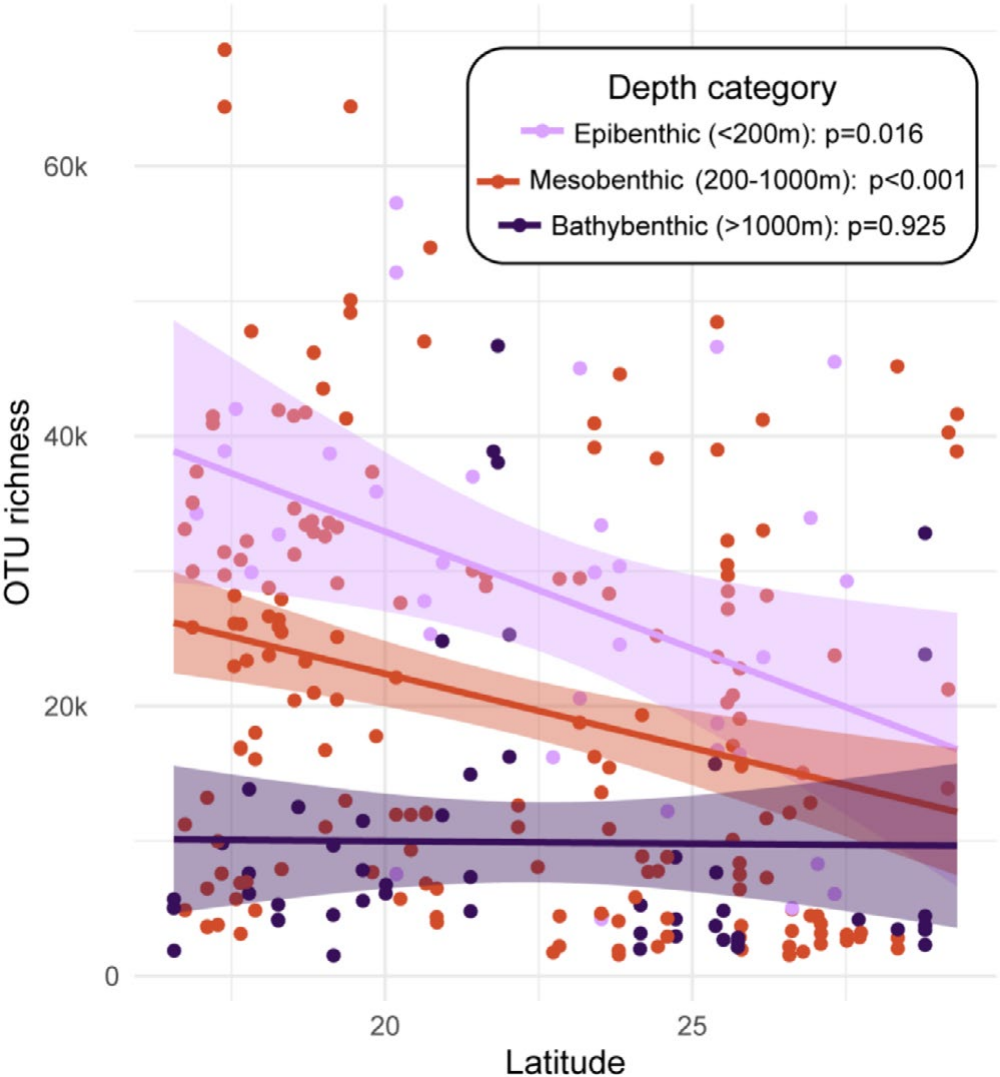
Relationship between OTU richness and latitude across depth categories, evaluated via linear regression models. *p* = statistical significance of the latitude coefficient for each depth category, indicating whether the observed relationship between OTU richness and latitude is likely due to chance; shaded areas: 95% confidence intervals.

OTU richness decreased in epibenthic (*p = 0.016*) and mesobenthic (*p < 0.001*) samples with latitude, with the greatest decrease observed in epibenthic samples (Figure 5). In contrast, bathybenthic communities showed no significant decrease in OTU richness with latitude (*p* = 0.924). This indicates that OTU richness of bathybenthic microbial communities is uniform across the Red Sea, while shallower communities show higher differences in OTU richness on a latitudinal gradient.

## 5 Discussion

### 5.1 Benthic Prokaryotic OTU Richness in the Red Sea

We detected a total of 287,613 OTUs, with a maximum of 98,600 OTUs observed at a single site and a median average OTU richness of 26,228 OTUs per site. Marine sediments are estimated to host between 32,800 and 2.46 million bacterial Amplicon Sequence Variants (ASVs) globally (Hoshino et al. 2020), suggesting that the diversity observed in our study aligns with the expected prokaryotic diversity in marine sediments. In contrast, a study investigating the pelagic bacterioplankton diversity of the Red Sea as part of the Red Sea Decade Expedition only found around 80,000 ASVs in total (Frühe et al. 2025), and while the authors used a different bioinformatics pipeline, which can impact the number of detected OTUs or ASVs, the observed bacterioplankton diversity of Frühe et al. (2025) is an order of magnitude lower than that of the present study, indicating that benthic prokaryotic diversity is much higher in the Red Sea than pelagic bacterioplankton diversity. This finding is consistent with previous global studies comparing pelagic and benthic beta diversity of bacteria (Lozupone and Knight 2007; Zinger et al. 2011) and is likely linked to greater habitat temporal stability, higher niche diversity and enhanced resource partitioning in marine sediments (Silvertown 2004).

### 5.2 Spatial Distribution of Benthic Prokaryotic Communities in the Red Sea

Our results reveal a clear stratification of prokaryotic communities by depth, with distinct differences among the epibenthic, mesobenthic and bathybenthic zones. Proteobacteria dominate marine sediments of the Red Sea, particularly the epibenthic zone, which is congruent with studies investigating marine sediments in other geographic regions (Bienhold et al. 2016; Walsh et al. 2016). In contrast, Acidobacteriota, Methylomirabilota and Gemmatimonadota are more prevalent in the bathybenthic zone. These depth‐related patterns align with previous studies showing that light availability, organic matter input, and redox conditions influence microbial community composition in marine sediments (Inagaki et al. 2015; Teske 2013).

OTU richness varied most in the epibenthic zone, characterised by higher sunlight exposure and more dynamic environmental conditions. Conversely, the OTU richness of bathybenthic microbial communities was uniform across the entire Red Sea, likely due to very stable environmental conditions at greater depths in the Red Sea (Klein et al. 2025; Naqvi 2021). This pattern was observed even though bathybenthic communities were sequenced at nearly twice the sequencing depth of epibenthic ones (Figure S3), indicating that their higher sequencing depth did not artificially inflate OTU richness.

While some studies have reported an increase in microbial richness with depth (Kembel et al. 2011; Pommier et al. 2010; Sunagawa et al. 2015; Walsh et al. 2016), our results support the conclusions of multiple other studies that documented the opposite trend (Agogué et al. 2011; Brown et al. 2009; Bryant et al. 2012). These contrasting conclusions indicate a complex relationship between microbial richness and depth that varies regionally. This relationship is further nuanced in the Red Sea due to the presence of a perennial oxygen‐depleted layer at varying mid‐depths (Naqvi 2021). Previous studies on microbial communities in oxygen‐depleted areas of the Red Sea (Klein et al. 2025) and other seas (Beman and Carolan 2013; Stevens and Ulloa 2008) reported a decrease in bacterial richness, demonstrating that other environmental factors need to be considered when analysing the relationship between microbial richness and depth.

Latitude also plays a crucial role in shaping microbial communities in the Red Sea, with distinct differences in alpha diversity and microbial community compositions observed across the latitudinal gradient. This pattern was persistent despite variations in sequencing depth among latitudinal Red Sea regions (Figure S2). These results align with the broader biogeographical patterns observed in the Red Sea, such as higher temperatures, lower oxygen, and lower salinity in the Southern Red Sea (Frühe et al. 2025; Trommer et al. 2009) and similar trends were observed in other marine ecosystems, where temperature and salinity gradients drive shifts in microbial diversity (Gilbert et al. 2009; Kirchman et al. 2010; Salazar et al. 2016).

Benthic prokaryotic communities in the Red Sea are also strongly influenced by oxygen availability. In particular, Chloroflexi exhibited a strong negative correlation with oxygen availability. This phylum has been detected in various anaerobic habitats, including sediments, hot springs and methanogenic anaerobic sludge (Speirs et al. 2019) and is a dominant phylum in the global deep ocean, exhibiting metabolic plasticity for survival under variable environmental conditions (Liu et al. 2022). Oxygen availability is a well‐known driver of microbial community composition in marine environments, with lower oxygen concentrations creating niches for unique bacterial taxa (Bandekar et al. 2024; Li et al. 2024; Stevens and Ulloa 2008; Wright et al. 2012). It should be noted, though, that only bottom‐water oxygen availability 1.5 m above the seafloor was measured as part of this study. Even though sediment samples were collected from the upper 1 cm of seafloor sediment, oxygen penetration into sediments is controlled primarily by sediment permeability, organic matter reactivity and bioturbation, and porewater oxygen in the top cm of sediments can differ markedly from bottom‐water concentrations (Glud 2008). Therefore, bottom‐water oxygen availability should be interpreted here as contextual rather than a direct measure of oxygen availability within the sediment.

### 5.3 Microbial Communities of Extreme Ecosystems

The extreme ecosystems sampled exhibit unique microbial communities distinct from their surrounding regions. For instance, the high abundance of Desulfobacterota in the Atlantis II brine pool highlights the specialised adaptations of these microbes to extreme conditions, such as high salinity and anoxia. These findings emphasise the importance of these unique environments as potential sources of unknown microbial diversity and metabolic capabilities (Varrella et al. 2020).

However, despite their unique environmental conditions, the extreme ecosystems sampled also show strong similarities in community composition with adjacent regions, suggesting a degree of connectivity and potential dispersal of microorganisms between these unique sites and the surrounding marine environment. This interplay between isolation and connectivity is a critical factor in understanding the ecological and evolutionary processes governing microbial diversity in extreme environments (Hamdan et al. 2013).

## 6 Conclusions

The unprecedented scope of the Red Sea Decade Expedition has yielded comprehensive insights into biogeographical patterns and environmental drivers of benthic microbial communities in the Red Sea. Given that the Red Sea is one of the warmest seas in the world, it serves as a model for investigating the impacts of climate change on marine biodiversity, particularly concerning coral reefs (Berumen et al. 2013; Fine et al. 2013). Our findings can aid in predicting climate change impacts on marine microbial diversity, especially in areas undergoing rapid environmental shifts. Expanding the community analysis presented here with metagenomic and functional gene analyses will help unravel the metabolic capabilities of key microbial taxa and their contributions to ecosystem functions. These insights will also expand our understanding of the resilience and adaptability of microbial communities in the Red Sea, particularly in the context of global climate change.

## Author Contributions

**Christopher A. Hempel:** methodology, software, formal analysis, investigation, data curation, writing – original draft, and visualization. **Larissa Frühe:** supervision, methodology, resources, investigation, and writing – original draft. **Sofia Frappi:** resources, writing – review and editing. **Elisa Laiolo:** resources, writing – review and editing. **Kah Kheng Lim:** resources, writing – review and editing. **Diego E. Rivera Rosas:** resources, writing – review and editing. **Amal A. Bajaffer:** resources, writing – review and editing. **Wajitha J. R. M. Sait:** resources, writing – review and editing. **Alexandra Steckbauer:** supervision, formal analysis, investigation, resources, writing – review and editing. **Taiba Alamoudi:** formal analysis, investigation, resources, writing – review and editing. **Jacqueline V. Alva García:** formal analysis, investigation, resources, writing – review and editing. **Shannon G. Klein:** supervision, formal analysis, investigation, resources, writing – review and editing. **Anieka J. Parry:** formal analysis, investigation, resources, writing – review and editing. **Mohammad A. Qurban:** conceptualization, project administration, supervision, funding acquisition, writing – review and editing. **Vincent A. Pieribone:** conceptualization, project administration, supervision, writing – review and editing. **Carlos Angulo‐Preckler:** supervision, resources, writing – review and editing. **Carlos M. Duarte:** conceptualization, project administration, funding acquisition, supervision, methodology, resources, writing – review and editing.

## Funding

This work was supported by the National Center for Wildlife and the Carlos Duarte.

## Conflicts of Interest

The authors declare no conflicts of interest.

## Supporting information


**Figure S1:** Number of filtered reads, that is, reads after bioinformatics processing, plotted against the number of OTUs per sample (after post‐clustering curation with LULU). The trendline is based on a nonlinear asymptotic saturation model fitted by nonlinear regression. The number of reads and OTUs are shown prior to removing negative controls with microDecon.
**Figure S2:** Mean sequencing depth per sample by Red Sea region. The number of reads is shown after removing negative controls with microDecon.
**Figure S3:** Mean sequencing depth per sample by depth category. The number of reads is shown after removing negative controls with microDecon.

**Table S1:** emi70216‐sup‐0002‐TableS1.csv.

**Data S1:** Supporting Information.

## Data Availability

The data that support the findings of this study are openly available in Bioproject PRJNA1216728 at https://www.ncbi.nlm.nih.gov/sra.
