# Supplementary material for: Assessing Prokaryotic Benthic Communities in the Red Sea
Source: Environ Microbiol. 2026 Feb 21;28(2):e70216. doi: 10.1111/1462-2920.70216 (PMC12924112; doi:10.1111/1462-2920.70216)
Supplement: Supplementary file 1 — Figure S1: Number of filtered reads, that is, reads after bioinformatics processing, plotted against the number of OTUs per sample (after post‐clustering curation with LULU). The trendline is based on a nonlinear asymptotic saturation model fitted by nonlinear regression. The number of reads and OTUs are shown prior to removing negative controls with microDecon. Figure S2: Mean sequencing depth per sample by Red Sea region. The number of reads is shown after removing negative controls with microDecon. Figure S3: Mean sequencing depth per sample by depth category. The number of reads is shown after removing negative controls with microDecon. [file EMI-28-e70216-s003.docx]

Supplementary Figures


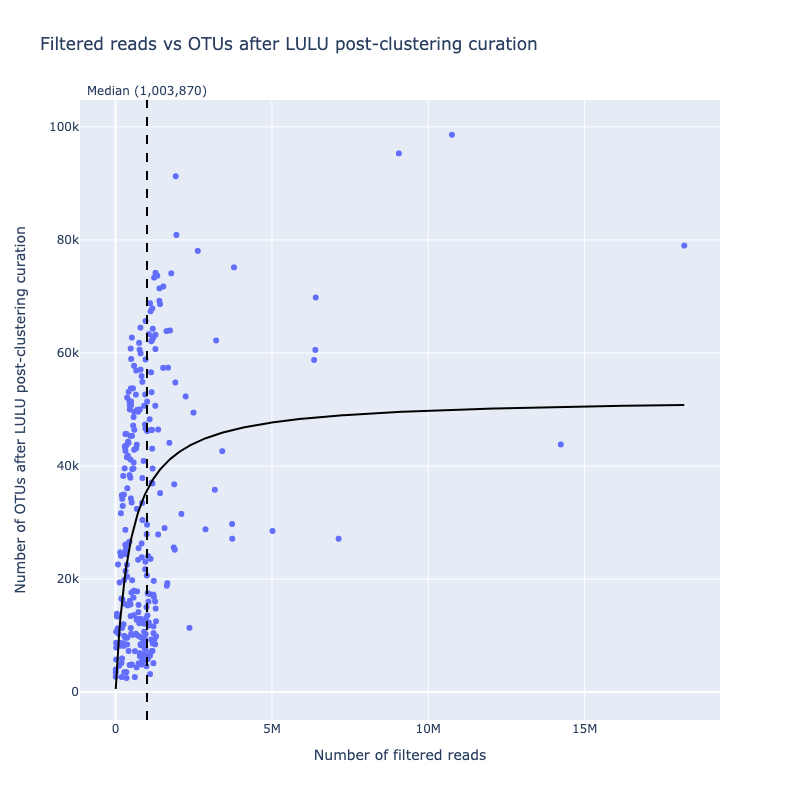


Supplementary Figure 1: Number of filtered reads, that is, reads after bioinformatics processing, plotted against the number of OTUs per sample (after post-clustering curation with LULU). The trendline is based on a nonlinear asymptotic saturation model fitted by nonlinear regression. The number of reads and OTUs are shown prior to removing negative controls with microDecon.


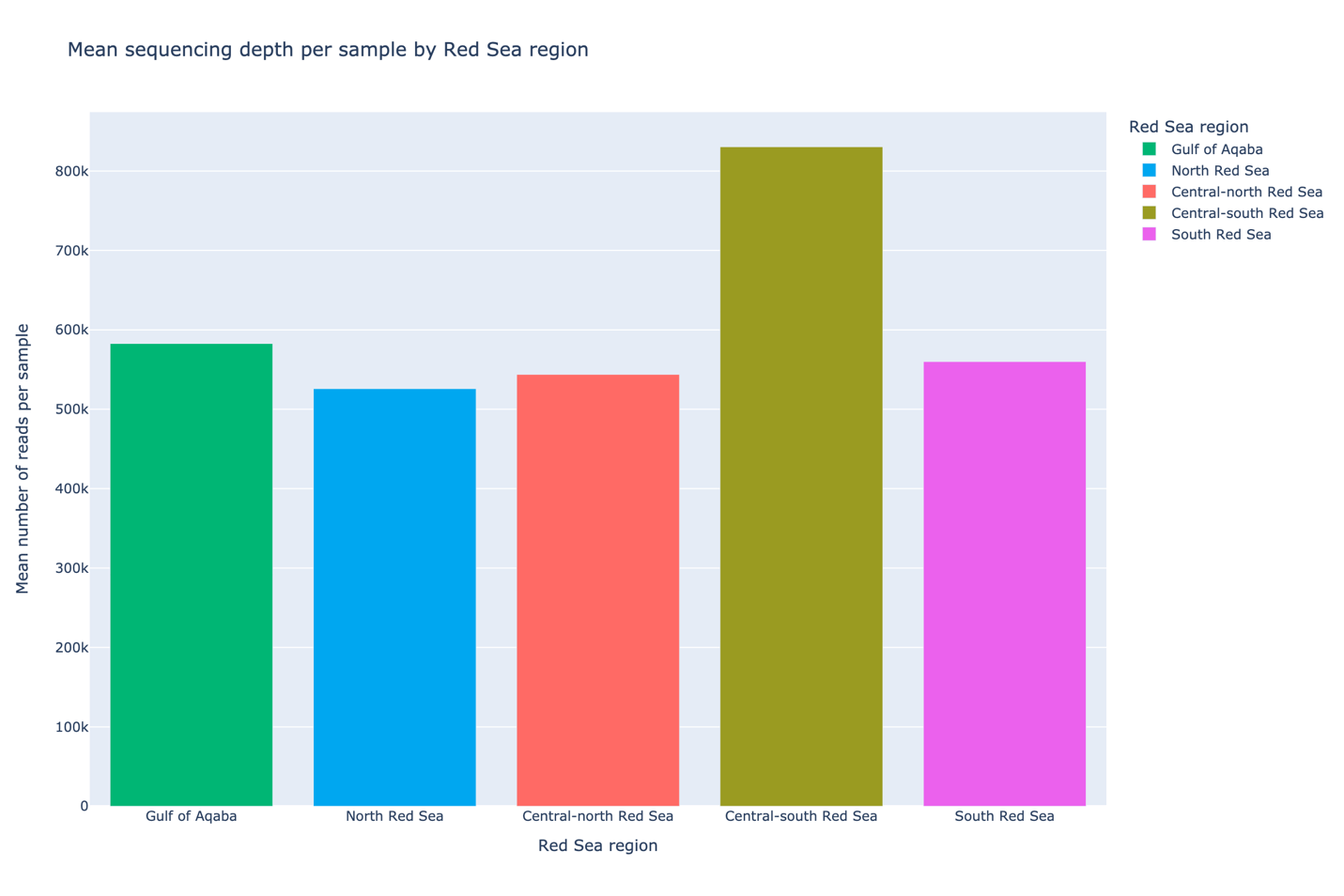


Supplementary Figure 2: Mean sequencing depth per sample by Red Sea region. The number of reads is shown after removing negative controls with microDecon.


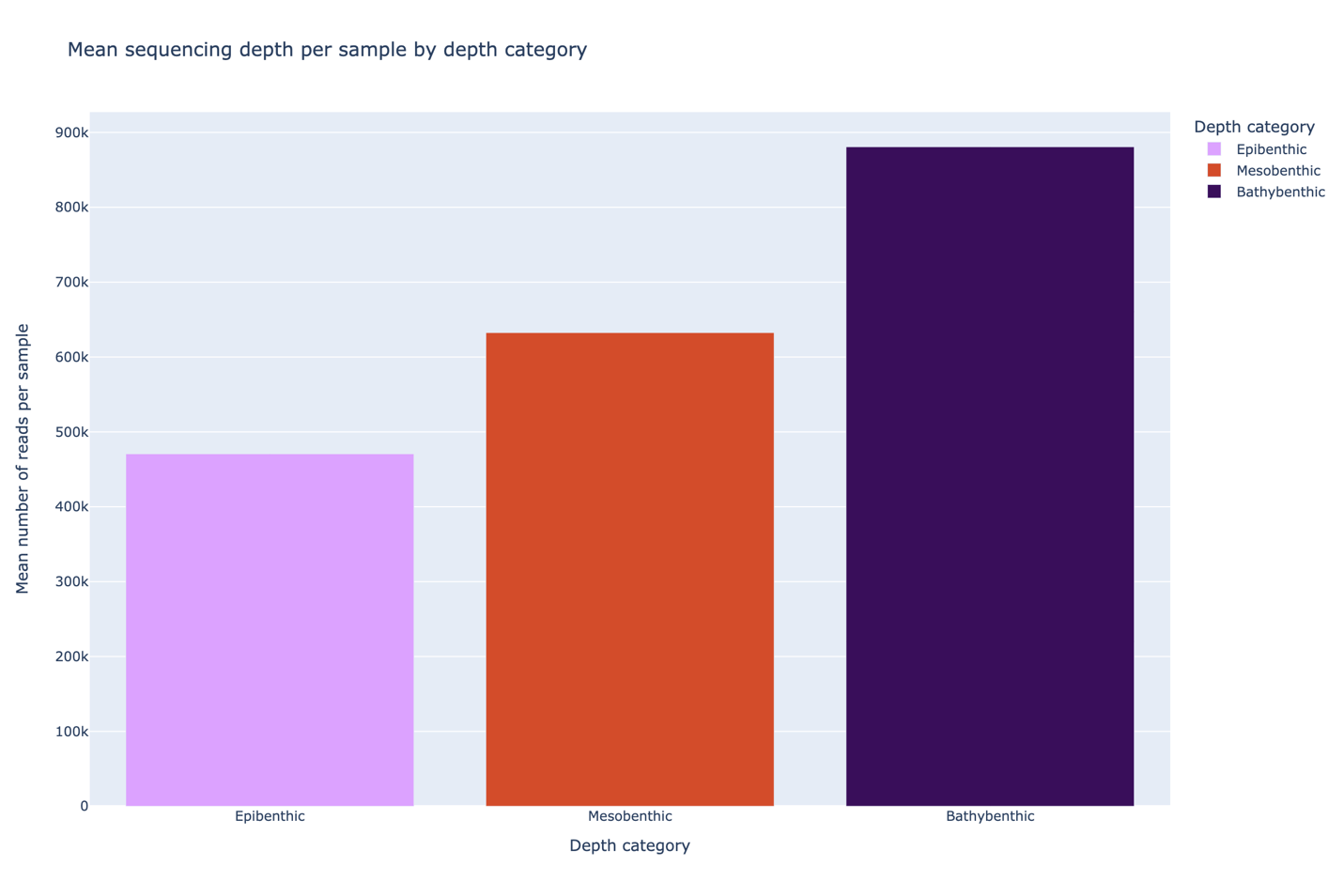


Supplementary Figure 3: Mean sequencing depth per sample by depth category. The number of reads is shown after removing negative controls with microDecon.
